# Metastasis of a transplantable mammary tumour in rats treated with cyclophosphamide and/or irradiation.

**DOI:** 10.1038/bjc.1977.181

**Published:** 1977-08

**Authors:** J. V. Moore, B. Dixon

## Abstract

**Images:**


*Br. J. Cancer* (1977) **36**, 221.

### METASTASIS OF A TRANSPLANTABLE MAMMARY TUMOUR IN RATS TREATED WITH CYCLOPHOSPHAMIDE AND/OR IRRADIATION

J. V. MOORE* AND B. DIXON

*From the Department of Radiotherapy, The University of Leeds, Cookridge Hospital, Leeds LS16 6QB*

Received 9 February 1977 Accepted 15 April 1977

**Summary.—We report observations on the spread by metastasis and infiltration of a transplantable tumour in rats treated by $^{60}$Co $\gamma$-irradiation of the primary, irradiation plus parenteral cyclophosphamide, or parenteral cyclophosphamide alone. The proportion of animals with overt disseminated disease and the extent of spread were measured with respect to the time elapsed after implantation and treatment of the primary tumour. The incidence of metastatic disease was broadly similar for all treatment groups, but the extent of dissemination was greater in rats whose treatment included cyclophosphamide.**

In the treatment of patients with cancer, the use of cytotoxic drugs as adjuvants to radiotherapy and surgery has become increasingly common (*e.g.* Choi and Carey, 1976). The probability of local control of a primary tumour may be increased by the independent cytotoxicity of these agents and by their modification of the response of malignant clonogenic cells to subsequent irradiation (Bleehen, 1973). We have carried out an experimental study on the response to combined treatment of a transplantable metastasizing mammary tumour in rats, using the alkylating agent cyclophosphamide (CP) in various combinations with radiotherapy (Moore, 1976). However, in clinical practice the major rationale for the adjuvant use of systemic chemotherapeutic agents has been their potential action on tumour metastases outside the irradiated volume (Tucker *et al.*, 1973; Roswit *et al.*, 1976). Whereas an "aggressive" regime of chemotherapy may favour destruction of metastases, it will also invariably produce a deleterious effect on the host (Slavin, Millan and Mullins, 1975). Should clonogenic cells of a metastasizing primary tumour survive a combined treatment, the environment into which they or their progeny are released may well differ from that prior to treatment. In our experimental mammary tumour, its propensity to metastasize was already known (Fig. 1). Accordingly, during studies of combined high-dose chemotherapy and irradiation of primary tumours, postmortem examination (PM) of rats was carried out routinely. The data obtained, which are reported in this paper, enable the incidence of local infiltration and distant metastasis and the extent of spread to be quantitated in relation to the treatment employed.

### MATERIALS AND METHODS

"John's strain" Wistar rats, sib-mated since 1939 (Thomlinson, 1960) were implanted with experimental tumours upon reaching a body weight of 150–200 g. Tumours were derived by serial transplantation from a spontaneous isogeneic mammary adenocarcinoma designated $LMC_1$. Details of the origin and growth characteristics of this tumour have been reported elsewhere (Moore and Dixon, 1977, in preparation). The chemotherapy and irradiation experiments from which data on infiltration and metastasis in 236 rats were derived were carried out with the 37th to 40th transplant generations, at

* Present address: Paterson Laboratories, Christie Hospital & Holt Radium Institute, Manchester M20 9BX.

FIG. 1.—Dissected female rat with an $LMC_1$ tumour of the 37th transplant generation. The animal was treated with cyclophosphamide i.p. and $\gamma$-irradiation to the primary (P). 90 days later, metastases were found in the ipsilateral and contralateral axillary (A), paraortic (Pa) and inguinal (I) lymph nodes. Metastases were more usually confined to ipsilateral nodes.

which stage the tumour was poorly differentiated and had a mean volume-doubling time of $4 \pm 1$ days. Tumours were implanted s.c. in the abdominal flank, as a pellet of tumour mince contained within a "sausage skin" of gut from a 50-g isologous rat (Thomlinson, 1960). Before implantation, the pellets were rinsed for 1 min in sterile distilled water to lyse tumour cells that might have adhered to their outer surface during preparation. Implanted pellets produced tumours which grew initially as encapsulated spheres unattached to skin or adjacent body wall and which attained within 10–12 days of implantation a mean diameter of 8–10 mm, at which size one of the following treatments was given:

1. Irradiation (500–7000 rad).
2. Cyclophosphamide (25–250 mg/kg body weight).
3. Cyclophosphamide (150 mg/kg) plus irradiation (1000–4000 rad).

Irradiations were given as single doses of $^{60}Co$ $\gamma$-rays, anaesthetized rats placed on a jig beneath lead shielding collimation, such that only the tumour and overlying skin were within the irradiation beam (Thomlinson and Craddock, 1967; Moore, 1976). The dose rate was 200 rad/min and scattered radiation to the body wall adjacent to the tumour and beyond was limited to 2% of the tumour dose.

All doses of CP (Endoxana; Ward Blenkinsop, Wembley) were administered in 0·9% saline as an i.p. injection. In the majority of cases treated by drug alone, CP was given in a single dose. In some instances a split dose (100 mg/kg $\times$ 2 q 1 to 10 days) was employed.

The major aim of all treatments was to produce delay in growth, not eradication of the primary tumour. Thus, all untreated and treated animals were killed by cervical dislocation when the primary tumour first reached a mean diameter of 35 mm, before it adversely affected the mobility of the host. The time taken to reach this size varied from 32 to 112 days after implantation, *i.e.* 20 to 100 days from treatment, according to the efficacy of treatment in delaying the growth of the primary. At PM, each animal was dissected and examined for infiltration of the tumour into adjacent tissues, and for the presence of macroscopic metastases in distant lymph nodes and abdominal and thoracic cavities. In the early experiments, gross evidence of tumour spread was confirmed by histological examination. For each animal, the PM was scored positive or negative for tumour spread, *i.e.* local infiltration or distant metastasis. Where positive, the sites of the body involved were recorded.

### RESULTS

Four major sites of tumour infiltration or distant metastasis were found (Fig. 2):

1. Infiltration of the abdominal body wall, followed by haemorrhage into the peritoneal cavity, adhesion and transcoelomic metastases to the peritoneum and diaphragm.
2. Metastases to the lungs.

FIG. 2.—Distribution of disseminated disease in animals found positive for tumour spread at PM. Data are from controls and all treatment groups. The number of occasions on which a particular site was scored positive is expressed as a percentage of the total number of positive sites observed (149). A—Invasion of body wall. B.—Metastases on peritoneum. C—Metastases on diaphragm. D—in thymus. E—in lungs. F—in axillary lymph nodes. G—in para-aortic lymph nodes.

3. Metastases to the ipsilateral axillary lympatic nodes.
4. Metastases to the ipsilateral para-aortic nodes.

Metastatic tumour was also found at other locations, *e.g.* the suprarenal lymphatics and thymus, but at a much lower frequency. Accordingly, subsequent analysis of data, for control animals and for treated groups, was confined to observations made at the 4 major sites listed above.

Overall, 45% of animals showed evidence of tumour spread at PM. More detailed analysis showed no direct correlation between incidence of positive PM and size of dose of CP or radiation, either alone or in combination. However, the variation in delay in growth of the primary due to the various treatments enabled a comparison of metastatic rate

TABLE.—*Absolute Numbers of Animals Examined for Gross Evidence of Tumour Spread, and Results of PM (+ or — for Spread). Intervals are those for the Primary Tumour to Grow from the Standard at Treatment (9 mm) to Killing (35 mm mean diameter) and thus are a Measure of the Efficacy of Treatment of the Primary.*

| Treatment | Control | | γ-alone | | CP-alone | | CP + γ | |
|---|---|---|---|---|---|---|---|---|
| (Days from treatment) | + | — | + | — | + | — | + | — |
| 20–29 | 3 | 13 | 0 | 8 | 1 | 15 | 1 | 0 |
| 30–39 | 0 | 3 | 5 | 10 | 13 | 24 | 6 | 3 |
| 40–49 | | | 6 | 8 | 17 | 18 | 8 | 3 |
| 50–59 | | | 6 | 8 | 2 | 0 | 3 | 4 |
| 60–69 | | | 9 | 5 | | | 2 | 1 |
| 70–79 | | | 6 | 3 | | | 7 | 0 |
| 80+ | | | 9 | 4 | | | 1 | 1 |

for an isoeffect on the primary, *i.e.* the time taken to reach 35 mm diameter (Table).

Thus data were analysed with respect to the time elapsed between tumour implantation, treatment and PM. This clearly showed (Fig. 3) that, overall, the incidence of positive PM increased with time, rising rapidly from about 10% at 30 to 40 days to about 70% at 80 to 100 days post-implantation. Because of the smaller quantity of data for analysis, interpretation by individual mode of

FIG. 3.—The proportion of animals with overt disseminated disease (positive PM) at increasing intervals after implantation of the primary tumours. ○—Untreated controls. ●—All treatment groups. Errors as s.d. calculated on the assumption of binomial distributions for presence or absence of tumour-spread at each interval.

FIG. 4.—The percentage of animals with overt disseminated disease at increasing intervals after treatment, calculated for the different treatments. ▪—untreated controls, □—γ-rays alone. ■—CP alone. ▨—CP+γ-rays. Figures above the bars give the total number of animals in each group examined for spread of tumour.

FIG. 5.—Mean number of sites affected in the body of animals revealing gross evidence of tumour spread at PM, calculated for increasing intervals after implantation and for different treatments. ●—γ-rays alone. □—CP alone. ○—CP+γ-rays. Errors as 2 × s.e., obtained from the number of affected sites in individual animals within a treatment group.

treatment was more difficult, but suggests (Fig. 4) that other than at the shortest time interval examined, *i.e.* after the least effective treatments, there was no significant difference in the incidence of animals with positive PM findings. For short delays in tumour growth, CP plus irradiation produced a significantly greater incidence than either chemotherapy or irradiation alone ($P<0\cdot05$).

In treated animals which were positive for tumour at PM, data were further analysed with respect to the total number of major sites in the body liable to be positive for disseminated growth (Fig. 5). In those animals in which the primary tumour was given radiation alone, the probability of tumour dissemination to more than one site increased only slowly with time. For rats treated by CP alone, the data are equivocal at the shorter time intervals, *i.e.*, the extent of metastatic spread is higher, but not significantly so, compared to irradiated animals. However, among those that were alive 50 days or more after CP plus irradiation (*i.e.* 50+12 days from tumour implantation), the extent of metastatic spread was significantly greater than after irradiation alone.

### DISCUSSION

The method of implantation used in this study probably militates against the early metastatic spread of tumour, possibly up to the time of treatment of the primary or shortly thereafter. However, this has yet to be tested by PM of animals after surgical removal of primary tumours at 8–10 mm diameter. Thus at this stage, explanation of mechanisms underlying the observed differences in the dissemination of $LMC_1$ tumour in rats given single or combined treatment are necessarily conjectural. Regardless of mechanisms, some of which are discussed below, the reported data underline the complexities of combination treatment of primary metastasizing tumours, when an aggressive first approach to chemotherapy may, in the event of failure, prejudice further attempts at cure of the disease.

If it is assumed that dispersion of $LMC_1$ tumour cells did occur shortly after implantation, their cycle time of 18 h (unpublished) would permit, in the 10–12 days required for the primary to reach treatment size, the establishment of occult metastatic foci each containing $6 \cdot 5 \times 10^4$ cells. Local irradiation of the primary tumour would not directly affect these but merely allow, by delaying its growth, the expression of overt metastatic disease as recorded at PM. Such irradiation may in fact enhance the growth of already existing metastases (van den Brenk and Sharpington, 1971; Sheldon and Fowler, 1973). Conversely CP, alone or in combination, should exert its cytotoxic effect on both the primary and metastatic foci. For the former, 150 mg/kg CP delays growth by approximately 15 days (Moore, 1976). One might therefore expect that such a dose would destroy, or at least markedly inhibit the growth of, metastases which contain far fewer cells of presumably equal or greater sensitivity to CP (Steel and Adams, 1975; Twentyman and Bleehen, 1976). Thus in drug-treated rats, the recorded incidence of disseminated disease should be initially lower than in rats with irradiated tumours. However, for short delays in growth of the primary (20–60 days), the incidence of positive PM was slightly higher in drug-treated than in irradiated rats, and with the shortest delay produced (20–40 days) was significantly higher when the drug was used in combination with radiation (Fig. 4).

If occult metastatic foci were not present at treatment, or the pretreatment contribution to spread was small, the data reflect wholly or mainly dissemination of clonogenic cells from the primary tumour in the conditions prevailing during or after treatment. Thus regimes that do not sterilize, but only produce an increasing delay in growth of the primary, would permit an increasing incidence of overt tumour spread, irrespective of the mode of treatment (Fig. 3). For drug alone or irradiation alone, this appears to have been the case, but at short intervals after combination treatment, the incidence was unexpectedly great (Fig. 4) and remained high thereafter, other than at 80–99 days for which results were obtained from only 2 animals. With this one exception, the incidence after CP or CP+$\gamma$ was as high or higher than that after irradiation alone and, at later times the extent of spread was markedly greater after the combination (Fig. 5). Others have speculated that such "potentiation" of dissemination by a cytotoxic agent might be attributed to suppression of an immunological response to tumour (Sugarbaker, Cohen and Ketcham, 1970; Brunner, Marthaler and Müller, 1971). Cyclophosphamide is a potent immunosuppressive agent, particularly at high dosage (Harris *et al.*, 1976).

The $LMC_1$ mammary tumour was of spontaneous origin and maintained in an inbred strain of rats. It has been claimed that specific immunological responses may sometimes be detected in animals bearing such tumours (Baldwin and Embleton, 1975). If so, the high initial incidence, and greater extent of spread of $LMC_1$ after combined treatment might be accounted for by a failure of surveillance. If, however, rejection responses to this kind of tumour do not occur (Hewitt, 1976) other explanations for the effect of CP must be sought; for example, greater lodgement of metastatic cells in tissues which have been subjected to non-specific trauma (Fisher, Fisher and Feduska, 1967).

This work was supported by Tenovus for whose generous financial assistance we express our thanks. We are grateful to Professor C. A. Joslin for his support and encouragement.

REFERENCES

BALDWIN, R. W. & EMBLETON, M. J. (1975) Immunology of Experimental Mammary Cancer. In *Host Defence in Breast Cancer*. Ed. B. A. Stoll. London: Heinemann. p. 78.

BLEEHEN, N. M. (1973) Combination Therapy with Drugs and Radiation. *Br. med. Bull.*, **29**, 54.

VAN DEN BRENK, H. A. S. & SHARPINGTON, C. (1971) Effect of Local X-irradiation of a Primary Sarcoma in the Rat on Dissemination and Growth of Metastases: Dose-response Characteristics. *Br. J. Cancer*, **25**, 812.

BRUNNER, K. W., MARTHALER, T. & MÜLLER, W. (1971) Unfavourable Effects of Long-term Adjuvant Chemotherapy with Endoxan in Radically Operated Bronchogenic Carcinoma. *Eur. J. Cancer*, **7**, 285.

CHOI, C. H. & CAREY, R. W. (1976) Small Cell Anaplastic Carcinoma of Lung: Reappraisal of Current Management. *Cancer, N.Y.*, **37**, 2651.

FISHER, B., FISHER, E. R. & FEDUSKA, N. (1967) Trauma and the Localisation of Tumour Cells. *Cancer, N.Y.*, **20**, 23.

HARRIS, J., SENGAR, D., STEWART, T. & HYSLOP, D. (1976) The Effect of Immunosuppressive Chemotherapy on Immune Function in Patients with Malignant Disease. *Cancer, N.Y.*, **37**, 1058.

HEWITT, H. B. (1976) Projecting from Animal Experiments to Clinical Cancer. In *Fundamental Aspects of Metastasis*. Ed. L. Weiss. Oxford: North-Holland. p. 343.

MOORE, J. V. (1976) The Response of a Rat Mammary Tumour to Cyclophosphamide and to Subsequent Irradiation. Ph.D. Thesis. University of Leeds.

ROSWIT, B., LIBERSON, S., OHANIAN, M., KUSIK, H. & PETROVICH, Z. (1976) Survival with Inoperable Lung Cancer. *N.Y. State J. Med.*, **76**, 560.

SHELDON, P. W. & FOWLER, J. F. (1973) The Effect of Irradiating a Transplanted Murine Lymphosarcoma on the Subsequent Development of Metastases. *Br. J. Cancer*, **28**, 508.

SLAVIN, R. E., MILLAN, J. C. & MÜLLINS, G. M. (1975) Pathology of High Dose Intermittent Cyclophosphamide Therapy. *Human Pathology*, **6**, 693.

STEEL, G. G. & ADAMS, K. (1975) Stem-cell Survival and Tumour Control in the Lewis Lung Carcinoma. *Cancer Res.*, **35**, 1530.

SUGARBAKER, E. V., COHEN, A. M. & KETCHAM, A. S. (1970) Facilitated Metastatic Distribution of the Walker 256 Tumour in Sprague–Dawley Rats with Hydrocortisone and/or Cyclophosphamide. *J. Surgical Oncol.*, **2**, 277.

THOMLINSON, R. H. (1960) An Experimental Method for Comparing Treatments of Intact Malign Tumours in Animals and its Application to the Use of Oxygen in Radiotherapy. *Br. J. Cancer*, **14**, 555.

THOMLINSON, R. H. & CRADDOCK, E. A. (1967) Gross Response of an Experimental Tumour to Single Doses of X-rays. *Br. J. Cancer*, **21**, 108.

TUCKER, R. D., SEALY, R., VAN WYK, C., LE ROUX, P. L. M. & SOSKOLNE, C. L. (1973) A Clinical Trial of Cyclophosphamide and Radiation Therapy for Oat Cell Carcinoma of the Lung. *Cancer Chemother. Rep.*, **4**, 159.

TWENTYMAN, P. R. & BLEEHEN, N. M. (1976) The Sensitivity to Cytotoxic Agents of the EMT6 Tumour *In vivo*. Comparative Response of Lung Nodules in Rapid Exponential Growth and of the Solid Flank Tumour. *Br. J. Cancer*, **33**, 320.


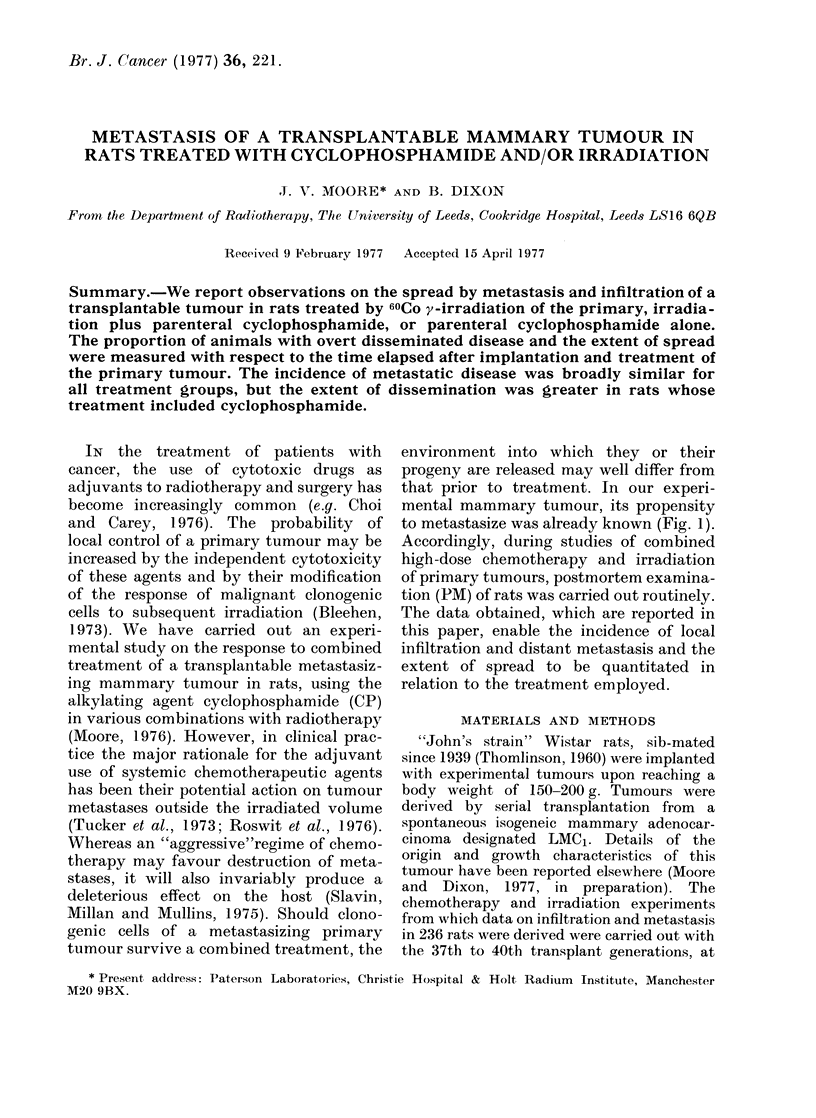

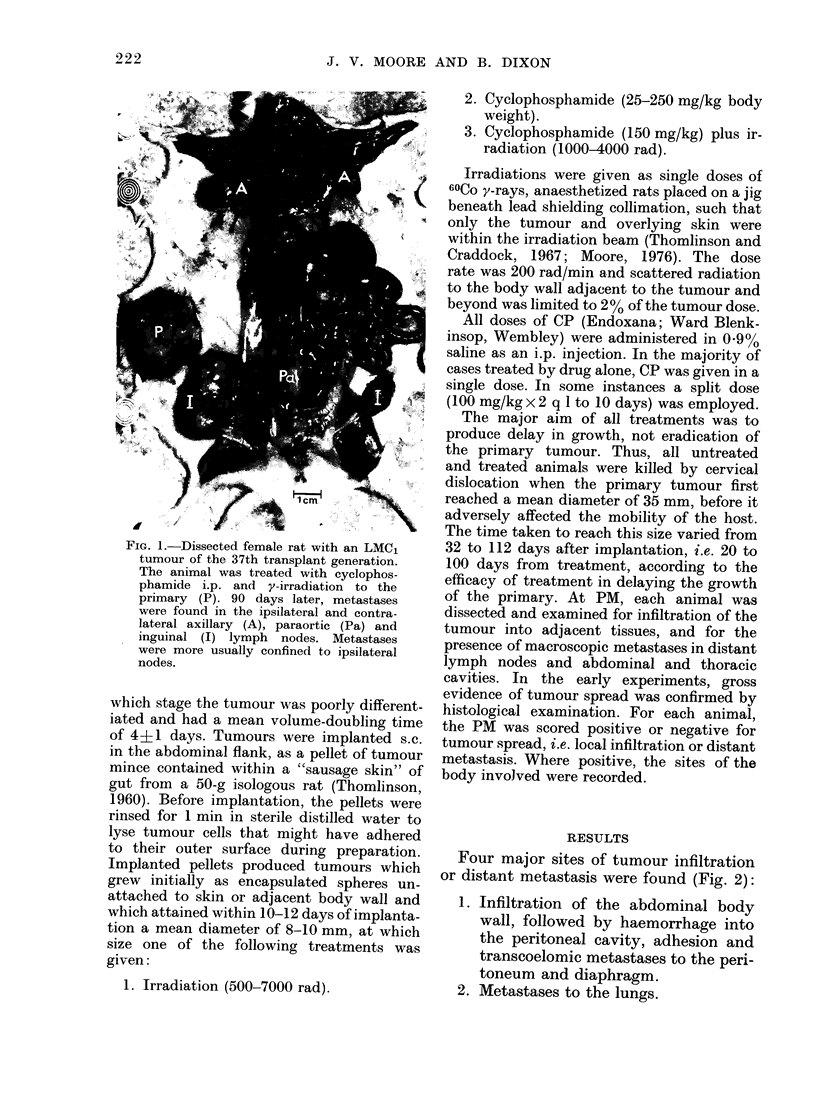

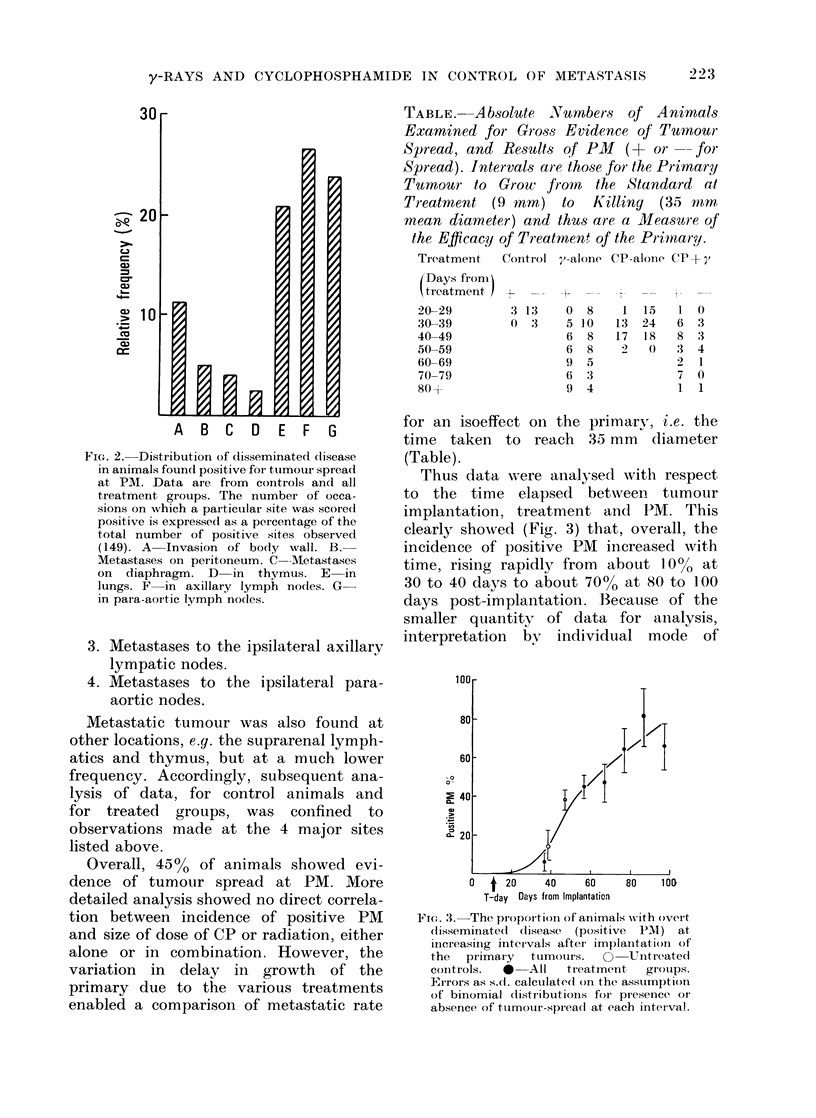

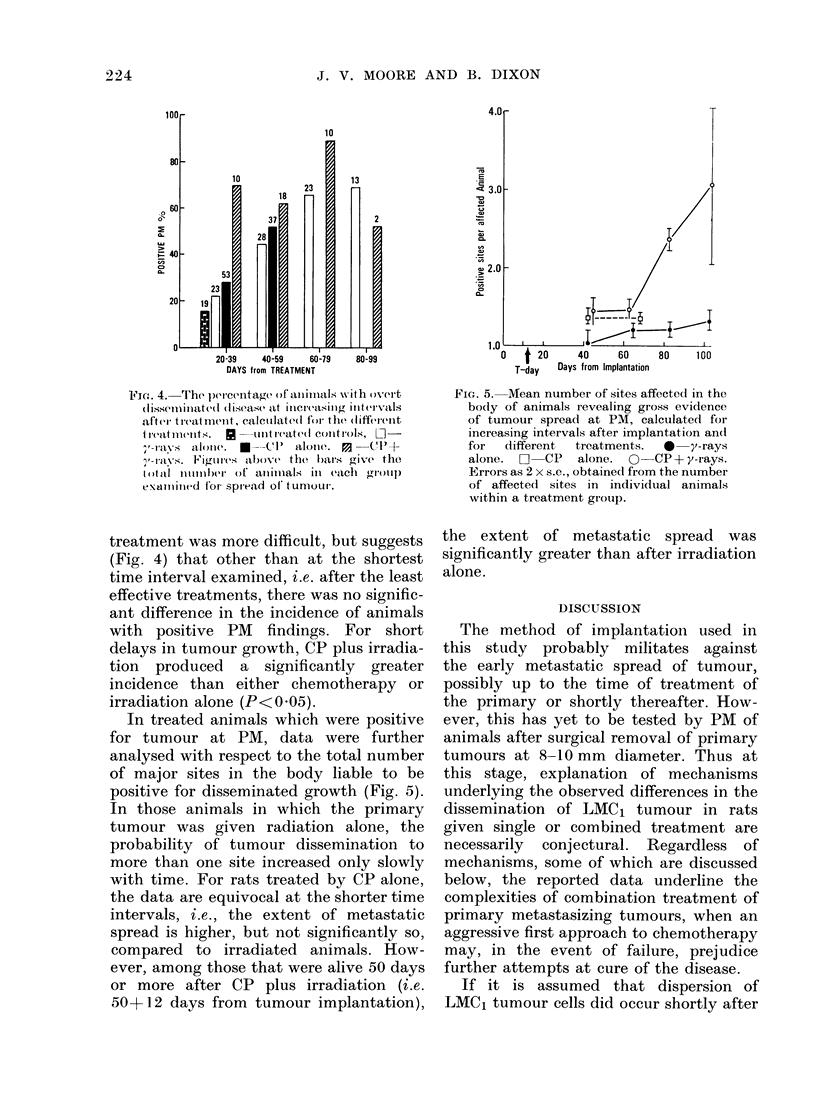

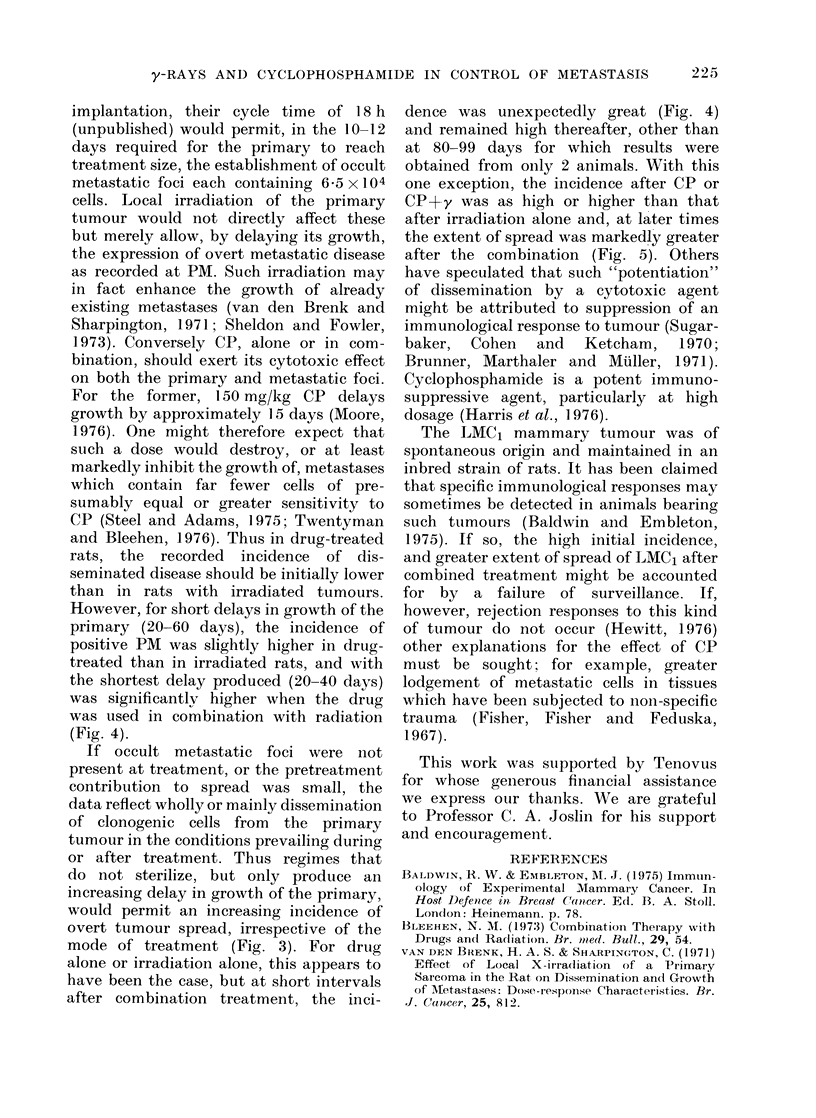

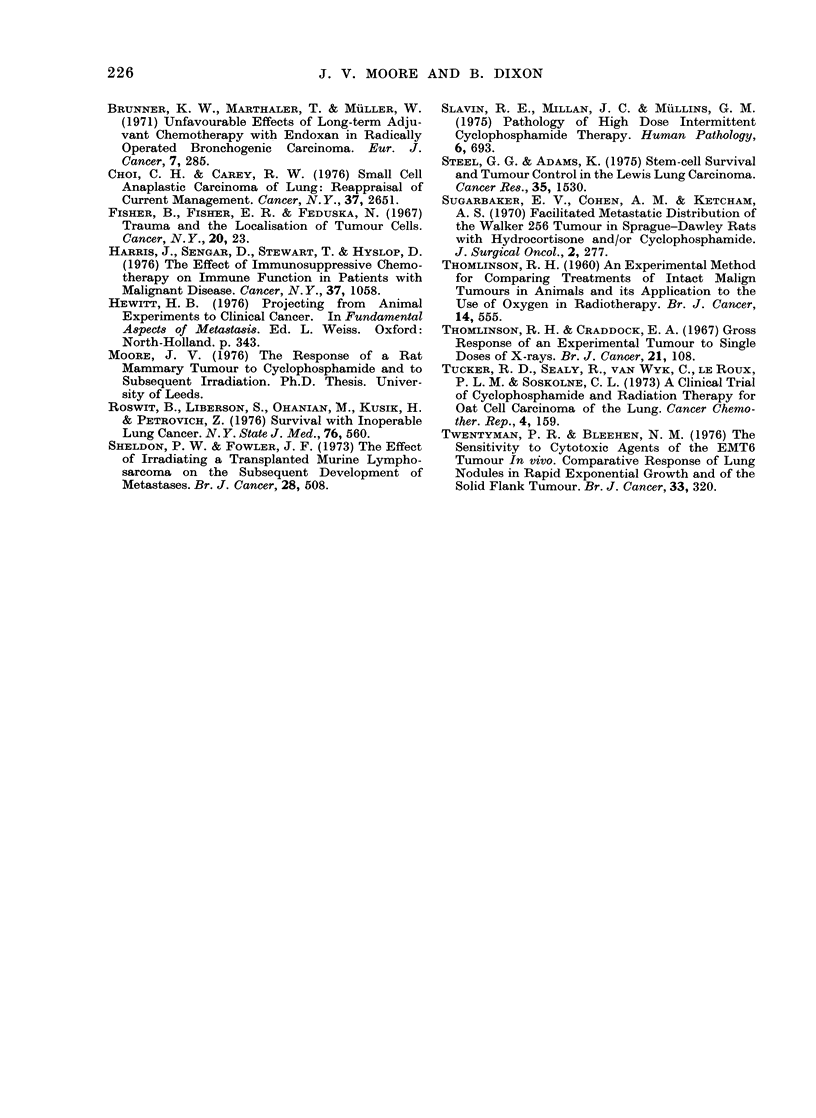


## References

[OCR_00584] Brunner K. W., Marthaler T., Müller W. (1971). Unfavourable effects of long-term adjuvant chemotherapy with Endoxan in radically operated bronchogenic carcinoma.. Eur J Cancer.

[OCR_00591] Choi C. H., Carey R. W. (1976). Small cell anaplastic carcinoma of lung. Reappraisal of current management.. Cancer.

[OCR_00596] Fisher B., Fisher E. R., Feduska N. (1967). Trauma and the localization of tumor cells.. Cancer.

[OCR_00601] Harris J., Sengar D., Stewart T., Hyslop D. (1976). The effect of immunosuppressive chemotherapy on immune function in patients with malignant disease.. Cancer.

[OCR_00619] Roswit B., Liberson S., Ohanian M., Kusik H., Petrovich Z. (1976). Survival with inoperable lung cancer. Controlled national Veterans Administration study of radiation and chemotherapy.. N Y State J Med.

[OCR_00624] Sheldon P. W., Fowler J. F. (1973). The effect of irradiating a transplanted murine lymphosarcoma on the subsequent development of metastases.. Br J Cancer.

[OCR_00630] Slavin R. E., Millan J. C., Mullins G. M. (1975). Pathology of high dose intermittent cyclophosphamide therapy.. Hum Pathol.

[OCR_00636] Steel G. G., Adams K. (1975). Stem-cell survival and tumor control in the Lewis lung carcinoma.. Cancer Res.

[OCR_00641] Sugarbaker E. V., Cohen A. M., Ketcham A. S. (1970). Facilitated metastatic distribution of the Walker 256 tumor in Sprague-Dawley rats with hydrocortisone and-or cyclophosphamide.. J Surg Oncol.

[OCR_00648] THOMLINSON R. H. (1960). An experimental method for comparing treatments of intact malignant tumours in animals and its application to the use of oxygen in radiotherapy.. Br J Cancer.

[OCR_00655] Thomlinson R. H., Craddock E. A. (1967). The gross response of an experimental tumour to single doses of x-rays.. Br J Cancer.

[OCR_00660] Tucker R. D., Sealy R., van Wyk C., le Roux P. L., Soskolne C. L. (1973). A clinical trial of cyclophosphamide (NSC-26271) and radiation therapy for oat cell carcinoma of the lung.. Cancer Chemother Rep 3.

[OCR_00667] Twentyman P. R., Bleehen N. M. (1976). The sensitivity to cytotoxic agents of the EMT6 tumor in vivo. Comparative response of lung nodules in rapid expotential growth and of the solid flank tumour.. Br J Cancer.

[OCR_00575] Van den Brenk H. A., Sharpington C. (1971). Effect of local x-irradiation of a primary sarcoma in the rat on dissemination and growth of metastases: dose-response characteristics.. Br J Cancer.

